# Effects of cannabidiol on vacuous chewing movements, plasma glucose and oxidative stress indices in rats administered high dose risperidone

**DOI:** 10.1038/s41598-022-24235-0

**Published:** 2022-11-16

**Authors:** Jaiyeola Abiola Kajero, Soraya Seedat, Jude Ohaeri, Abidemi Akindele, Oluwagbemiga Aina

**Affiliations:** 1 Department of Psychiatry, Faculty of Medicine and Health Sciences, Stellenbosch University, Francie van Zijl Drive Tygerberg, PO Box 241, Cape Town, 7505 South Africa; 2 Department of Psychological Medicine, Teaching Hospital, University of Nigeria, P.O. Box 3236, Enugu, Enugu State Nigeria; 3 Department of Pharmacology, Therapeutics & Toxicology, Faculty of Basic Medical Sciences, College of Medicine, University of Lagos, Private Mail Bag 12003, Lagos, Nigeria; 4 Department of Biochemistry and Nutrition, Nigerian Institute of Medical Research, 6 Edmund Crescent, Off Murtala Mohammed Way, P.M.B. 2013, Yaba, Lagos, 100001 Nigeria; 5 Present Address: Federal Neuropsychiatric Hospital, 8, Harvey Road, P.M.B 2008, Yaba, Lagos Nigeria

**Keywords:** Biochemistry, Neuroscience, Medical research

## Abstract

Atypical antipsychotics, despite their rapid dissociation from dopamine receptors and reduced tendency to induce oxidative stress, have been associated with difficult-to-manage movement disorders, including tardive dyskinesia (TD). The study set out to investigate the effects of cannabidiol (CBD), a potent antioxidant, on risperidone-induced behavioural and motor disturbances; namely vacuous chewing movements (VCM), and oxidative stress markers (e.g. superoxide dismutase (SOD), catalase (CAT), reduced glutathione (GSH), malondialdehyde (MDA), Nitric oxide (NO), and DPPH (2,2-diphenyl-1-picrylhydrazyl)). Oral risperidone (10 mg/kg) or oral CBD (5 mg/kg) were administered to six experimental groups. While risperidone alone was administered for 28 days, CBD concomitantly or in sequential order with risperidone, was administered for 28 days; and CBD alone was administered for 21 days. Behavioural, motor, and specific biochemical parameters, which included VCM, muscle tone, fasting blood sugar (FBS), and oxidative stress markers were assessed at different time points after the last dose of medication. Oral CBD (5 mg/kg) significantly reduced risperidone-induced elevated FBS when given after the administration of risperidone. Oral CBD also had effects on VCM when administered before risperidone and similarly, attenuated risperidone-induced increased muscle tone. It was also established that concomitant or sequential administration of CBD and risperidone did not have any adverse effects on cognition or locomotion. Both CBD and risperidone increased the activity of antioxidant enzymes and decreased the activity of pro-oxidant enzymes. This study suggests CBD could mitigate metabolic dysregulation and extrapyramidal side effects associated with risperidone without producing cognitive impairments.

## Introduction

The reintroduction of clozapine to the market in 1989 after the first attempt in 1970^1,2^ heralded a new generation of antipsychotics referred to as atypical antipsychotics (AAPs) or second-generation antipsychotics, which include clozapine, olanzapine, risperidone, quetiapine, lurasidone, and ziprasidone among others. These antipsychotics are called atypical because of their serotonin 5-HT_2A_ receptor antagonism in combination with D_2_ receptor antagonism properties^3,4^. Initially, they were believed to carry a lower burden of extrapyramidal side effects (EPS) such as parkinsonism, acute dystonia, akathisia, and akinesia compared to the typical or first-generation antipsychotics; but more recent studies have disagreed with this assertion. Parkinsonism, for example, has been observed to increase in frequency with high doses of atypical antipsychotics^5–7^. In addition to EPS, antipsychotics have significant metabolic side effects like weight gain, diabetes mellitus, and hyperlipidaemia which can increase the risk of cardiovascular mortality^8–10^.

Apart from the acute motor disorders, other movement disorders associated with atypical antipsychotics include neuroleptic malignant syndrome, tardive dystonia, and tardive dyskinesia (TD)^11,12^. Tardive dyskinesia is a late-onset movement disorder that is potentially irreversible. Though the pathophysiology is not yet clearly elucidated, dopamine receptor upregulation and supersensitivity, dysfunction of other neurotransmitters such as glutamate, γ-amino butyric acid (GABA), serotonin, neurotoxicity, oxidative stress, changes in synaptic plasticity, defective neuroadaptive signalling, and defective antipsychotic metabolising enzymes have been implicated in the aetiology^13–15^. Risk factors for TD include duration of antipsychotic use, age, genetics, and ethnicity^16–19^.

It was initially thought that the incidence of TD would decline with increasing use of AAPs, because of the rapid dissociation of most AAP molecules from receptor sites, reduced tendency to induce oxidative stress, and the ability of most AAPs to bind 5HT_2_ receptors thereby reducing the tonic effects of dopamine in the basal ganglia^20–22^. Despite a 30% reduction in the prevalence of TD with AAPs compared with typical antipsychotics^23,24^, the rate of 20% of TD with AAP is still high.

While AAP-induced movement disorders have been observed, there appears to be little or no association with oxidative stress indicators^25–27^. Some authors have also suggested that AAPs, following chronic administration, can reduce pro-oxidants and increase GSH^28^. Another study observed an increase in antioxidant defence enzymes and lipid peroxidation in rat brains after chronic haloperidol administration but there was no change after chronic risperidone, clozapine, and olanzapine administration^25^. The mechanisms by which AAPs induce movement disorders, such as TD, remain a puzzle considering that these disorders have been associated with an imbalance between pro-oxidants and antioxidants in various studies^17,29–31^. It is therefore important to investigate the association between VCM, AAPs, antioxidants, and pro-oxidants in an animal model of TD using risperidone as the prototype APP.

Though the mechanism by which AAPs influenced oxidative stress is poorly understood, AAPs have been observed to alleviate microglia-induced oxidative stress by reducing free radical production in the microglia^32^. Risperidone has been postulated to reduce reactive oxygen species (ROS) production through the regulation of antioxidant enzymes in the brain^33^. In contrast, risperidone has been shown to induce oxidative stress in the liver through increased reactive oxygen species (ROS) production, thus leading to mitochondrial collapse, lysosomal membrane disruption, GSH depletion and lipid peroxidation^34^. Pancreatic damage, including pancreatitis, is also associated with risperidone^35–37^, and oxidative stress may play a role here as pancreatitis is associated with oxidative stress^38–40^. The damage to pancreatic β cells following AAP-induced glucose intolerance may be related to increased free radical production^41,42^. In addition, amelioration of oxidative stress through the administration of a potent antioxidant may also reduce the risk of metabolic syndrome and improve medication adherence and overall quality of life. Identifying an ideal potent antioxidant that can alleviate the side effects of the AAP is an avenue of research that is worth pursuing.

There is much interest in Cannabidiol (CBD), a non-psychoactive and non-reinforcing cannabis sativa constituent^43,44^, which several studies have confirmed to have anti-oxidative, anti-inflammatory, and neuroprotective properties^45,46^. It has also shown promise in the prevention and treatment of movement disorders, including tardive dyskinesia, in animal models^47,48^. Furthermore, it is devoid of any major adverse reactions in both animal and human studies^49–51^, thus making CBD the ideal antioxidant for this study. CBD may, however, be similar to AAPs in its pharmacological profile because in animal models it has antipsychotic-like effects without inducing extrapyramidal-like effects^52^.

Meanwhile, clinical studies on the glycaemic effects of CBD have yielded conflicting results as one study observed an improvement in insulin secretion while another did not observe an improvement in glycaemic control^53^. As a best practice, it is prudent and essential to study the effects of CBD in combination with AAPs using risperidone as a prototype to affirm if synergistic actions of the two compounds (CBD and risperidone) can mitigate adverse effects, especially cognitive and metabolic side effects. Our aim was to determine if CBD can ameliorate risperidone induced VCM and reduce the metabolic side effects of risperidone. Our secondary objective was to explore the association between risperidone-induced VCM and oxidative stress indices.

## Materials and methods

### Animals

Male Wistar adult rats used in this study were obtained from the colony of the Nigerian Institute of Medical Research (NIMR), Yaba, Lagos, Nigeria. The animals were kept in clean polypropylene cages in well-ventilated, and hygienic compartments maintained under standard environmental conditions. The rats were also fed with standard rodent pellets (produced by Ladokun Feed Plc, Ibadan, Nigeria), and with water ad libitum*.* The animals were acclimatized for a period of 2 weeks before experimental procedures were undertaken in accordance with the United States National Institutes of Health Guidelines for Care and Use of Laboratory Animals in Biomedical Research^54^. This study was also approved by the Institutional Review Board (IRB) of the Nigerian Institute of Medical Research, Yaba, Lagos, Nigeria (IRB/16/329) and Stellenbosch University’s Health Research Ethics Committee: Animal Care and Use (SU-ACUD16-00137).

### Drugs

Cannabidiol [(–)-Cannabidiol, GMP (Cannabidiolum)] (CBD) (VAKOS X, a.s., Permova 28a, 186 00 Praha 8, in Czech Republic. Company number: 04801938). The drug was supplied in fine granule form with the amount administered weekly calculated and dissolved in 70% ethanol as recommended by the manufacturers. It was also diluted with distilled water. CBD was administered orally. Risperidone tablets (manufactured by Janseen Pharmaceuticals Beerse, Belgium) were dissolved in distilled water and administered orally.

### Experimental design

The experimental groups (*n* = 51) consisted of six groups: A (*n* = 10), Group B (*n* = 9), Group C (*n* = 8), Group D (*n* = 8), Group E (*n* = 8) and Group F (*n* = 8).

Pharmacological administration was as follows: Group A (Control (Distilled water) 2 ml p.o.), Group B (Oral CBD 5 mg/kg/oral), Group C (Risperidone 10 mg/kg/oral), Group D (CBD 5 mg/kg/oral + Risperidone 10 mg/kg/oral), Group E (Risperidone 10 mg/kg/oral then CBD 5 mg/kg/oral), Group F (CBD 5 mg/kg/oral then Risperidone 10 mg/kg/oral). Some researchers used 10 mg/kg of risperidone to induce VCM^55^. According to some investigators, effective doses of CBD in rats range between 2.5 and 10 mg/kg^56^.

In Group D, CBD was administered once daily for 28 days, while risperidone was administered concomitantly but in divided doses for 28 days. In Group C, Risperidone 10 mg/kg/oral only was administered daily in divided doses for 28 days, while administration for Groups A (Control (Distilled water only) 2 ml p.o.) and B (Oral CBD 5 mg/kg/oral only) was once daily for 21 days. In Group E, Risperidone 10 mg/kg/oral was administered for 28 days before the administration of CBD 5 mg/kg/oral for 28 days and in Group F, CBD (5 mg/kg/oral) was administered for 28 days before the administration of risperidone (10 mg/kg/oral) for 28 days. VCM was assessed at 8 h, 24 h, and on Day 8 after the last dose of the medication. Day 8 VCM was used in the analysis because persistent VCM after withdrawal of medications is the appropriate model of TD, though VCM observed after Day 8 have been shown to persist for a long time^55^.

The rats were fasted overnight after the last meal at 7 pm until 7 am when blood was taken for FBS measurement. A fine sterile disposable hypodermic needle was used to puncture the rats at the end of their tails, and enough blood was dropped on a portable glucometer device, Accu-Chek Performa^®^ (Roche Diagnostics, Mannheim, Germany), to obtain a FBS reading.

For all groups, weight and FBS were measured the day before administration of medication and a day after administration of the last dose of medication.

Notably, risperidone-induced FBS changes in rats have been shown to be stable over time^57^.

### Behavioural assays

Elevated plus maze, open field, rota-rod, and object recognition tests were used to assess behavioural responses. For all groups, behavioural assessments were carried out 24 h after the last dose of medication or distilled water (control group).

VCM were assessed by placing each animal in individual transparent glass plexiform cages. Each animal was allowed to acclimatize for 5 min before counting started. The number of VCM (mouth openings in the vertical plane not directed toward physical material) was counted for 10 min^58^. Day 8 VCM was used in the analysis because persistent VCM after withdrawal of medications is the appropriate model of TD; though VCM observed after Day 8 have been shown to persist for a long time^55^. Methodologies used in behavioural assays were described in detail in our previously published study^48^.

#### Elevated plus-maze test

Anxiety was monitored using this test^59^. The elevated plus-maze consisted of two open arms (30 × 5 cm), and two closed arms (30 × 5 × 15 cm) that extended from the central platform (5 × 5 cm). The entire maze was elevated to 40 cm above the floor. During the first 5 min of free exploration, the number of entries into and the time spent in the open and closed arms were recorded. An entry was defined as the point at which the animal placed all four paws onto the arm.

#### Open-field test

This test was used to assess locomotion in the study^60,61^. The number of line crossings is used as a measure of locomotor activity. The number of line crosses and the frequency of rearing are usually used as a measure of locomotor activity, exploration, and anxiety. A high frequency of these behaviours indicates increased locomotion and exploration and/or a lower level of anxiety^60^. The open-field box is a rectangular area composed of a hard floor measuring 36 cm × 36 cm × 26 cm and made of a white painted wood. The floor was divided by permanent red markings into 16 equal squares at the bottom. Each rat was introduced singly into one corner of the field and after each session, the area was cleaned with 70% alcohol to eliminate olfactory bias, and the area was thereafter allowed to dry before introducing a new animal. The locomotor activity was measured for 10 min. A high frequency of these behaviours indicates increased locomotion^60^.

#### Object recognition test

This test was used to study drug-induced cognitive effects^62^. Each animal was placed in a plexiform glass transparent cage and was allowed to acclimatise for 5 min before two similar spherical objects (Balls) were introduced. The animal was allowed to spend 10 more minutes in the cage with the objects and the time the animal spent exploring the objects was measured with a stopwatch by an observer before the animal was removed. The animal was re-introduced into the cage after an hour, and a different object (square shaped) was added into the cage before the animal was re-introduced, while the previous objects (Balls) were removed. Time spent exploring both new and old objects was measured for 10 min by two different observers using stopwatches. The time spent by the animal using its nose to touch the object was measured while the time spent using other parts of the body were ignored.

#### Rota-rod test

The method was used for the assessment of motor coordination, balance, and grip strength in rodents^63^. A rota-rod treadmill device (Ugo Basile No. 7600 Varese, Italy) was used for this purpose. The rats were trained to remain on slowly moving (16 revolutions min^−1^) rods of 5 cm in diameter for 150 s by walking. The animals were then placed on the treadmill after training and the time spent on the treadmill before falling was measured for each animal.

### Antioxidant indices assays

Animals were sacrificed on Day 8 after the administration of the last dose of pharmacological agent by first anesthetizing them with phenobarbitone before cervical dislocation. Methodologies used in determining antioxidant indices were also described in detail in our previously-published study^48^. The following antioxidant indices were determined spectrometrically.

#### Malondialdehyde (MDA)

MDA is an index of lipid peroxidation, and it was determined using the method of Buege^64^.

#### Reduced glutathione (GSH)

The GSH content of the brain as non-protein sulfhydryl was estimated according to the method described by Sedlak and Lindsay^65^.

#### Catalase (CAT)

CAT activity was determined according to the method of Sinha^66^.

#### Superoxide dismutase (SOD)

SOD activity was determined as described by Sun and Zigma^67^.

#### Nitric oxide (NO) scavenging activity

This was measured by the Griess reagent assay.

#### DPPH scavenging assay

This was determined using the methods described by Kedare and Singh^68^.

### Statistical analysis

Descriptive statistics of relevant study variables (weight, FBS, VCM, and behavioural and biochemical assays) are presented using measures of centrality and dispersion (mean and standard deviation). The normality of distribution of the data was evaluated using histograms and supported by the results of the Kolmogorov–Smirnov test. Comparison of means was done using one way-ANOVA. When group differences were statistically significant (p-value < 0.05), pairwise comparisons or post-hoc tests were done. Omnibus tests were also used with post-hoc comparisons. Bonferroni corrections were used to adjust for multiple comparisons when necessary.

### Statement on reporting experimental procedures

Reporting in this study follows the recommendations in the ARRIVE guidelines.

### Ethical statement

The study was approved by the Institutional Review Board (IRB) of NIMR, Yaba, Lagos, Nigeria (IRB/16/329) and Stellenbosch University’s Health Research Ethics Committee: Animal Care and Use (SU-ACUD16-00137).

## Results

### Effects of interventions on fasting blood sugar (FBS) and weight gain

Before the administration of medications, there was a statistically significant difference in FBS between groups (F = 14.337, p < 0.001, df = 5). On post-hoc analysis, there was a significant between-group difference in FBS pre-medication in Groups D and E (p < 0.001), Groups C and E (p < 0.001), Groups E and F (p < 0.001), Groups E and A (p < 0.001), Groups E and B (p = 0.001), and Groups F and B (p = 0.016).

**Figure 1:**
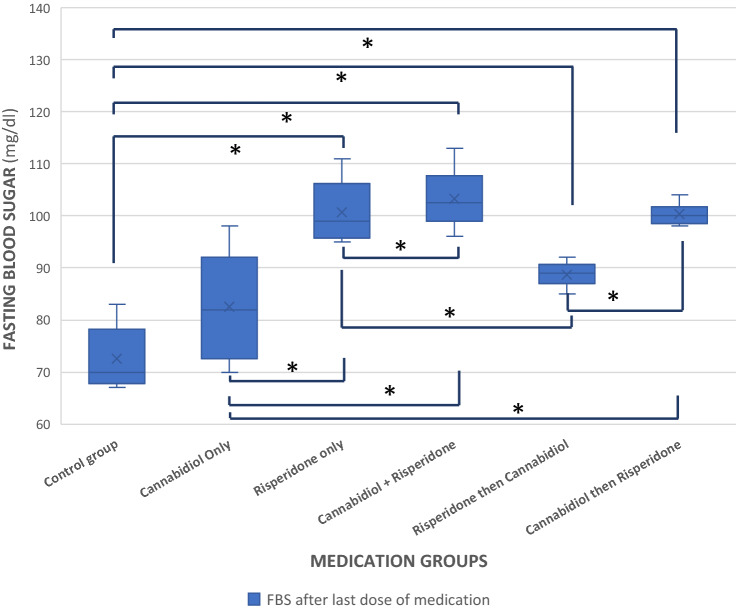
Fasting blood sugar across groups after the last dose of medications. Observations of the post-pharmacological interventions FBS values in each group indicated CBD influenced the FBS values. *Statistically significant difference.

Similarly, after the last dose of medication administration, there was a statistically significant difference in the mean FBS between groups (F = 34.079, p < 0.001, df = 5). On post-hoc analysis, there was a significant difference in FBS between Groups D and E (p = 0.009), D and A (p < 0.001), D and B (p < 0.001), C and E (p = 0.004), C and A (p < 0.001), C and B (p = 0.006), E and F (p < 0.001), E and A (p < 0.001), F and A (p < 0.001), F and B (p = 0.006) (Fig. 1).

There was no statistical difference in mean weight before (F = 0.469, p = 0.797, df = 5) and after (F = 2.837, p = 0.062, df = 5) the last dose of administration of pharmacological agents. After the last dose of medications, subjects in Group D had the highest mean weight gain (45.5 ± 40.0), while those in Group F had the lowest mean weight gain of the six groups (3.2 ± 38.4). However, there was no statistically significant difference in the weight between the groups (F = 1.831, p = 0.127, df = 5) (Table 1).

**Table 1:** Mean weight gain, object recognition, and locomotor activity after the last dose of pharmacological intervention. Significant values are in bold.

Variable	Mean ± SD	F	p-value		Mean ± SD	F	p-value
**Mean weight gain**				**Discrimination Index**			
Group A	3.2 ± 38.4	1.831 df = 5	0.127	Group A	0.7 ± 0.2	1.611 df = 5	**0.178**
Group B	10.8 ± 17.8			Group B	0.3 ± 0.6		
Group C	33.4 ± 35.1			Group C	0.5 ± 0.3		
Group D	45.5 ± 40.0			Group D	0.3 ± 0.3		
Group E	13.8 ± 35.4			Group E	0.7 ± 0.4		
Group F	35.4 ± 42.4			Group F	0.5 ± 0.5		
**VCM**				**ROTA ROD**			
Group A	0.3 ± 0.5	9.131 df = 5	0.000	Group A	62.7 ± 35.6	**2.738** df = 5	**0.031**
Group B	0.0 ± 0.0			Group B	63.9 ± 24.2		
Group C	4.8 ± 3.5			Group C	23.8 ± 21.9		
Group D	2.5 ± 1.4			Group D	94.2 ± 34.4		
Group E	2.3 ± 1.6			Group E	35.1 ± 23.3		
Group F	1.0 ± 1.0			Group F	77.0 ± 82.9		
**Calculated open arm**				**Line crossing**			
Group A	10.0 ± 0.5	0.334 df = 5		Group A	5.9 ± 4.3	2.262 df = 5	0.092
Group B	11.1 ± 3.8			Group B	5.9 ± 5.9		
Group C	12.5 ± 2.0		0.890	Group C	15.1 ± 14.6		
Group D	16.7 ± 0.8			Group D	2.8 ± 1.3		
Group E	12.5 ± 0.6			Group E	10.1 ± 5.9		
Group F	12.5 ± 1.0			Group F	8.5 ± 7.9		

### Behavioural assays after the last dose of pharmacological interventions

#### VCM

**Figure 2:**
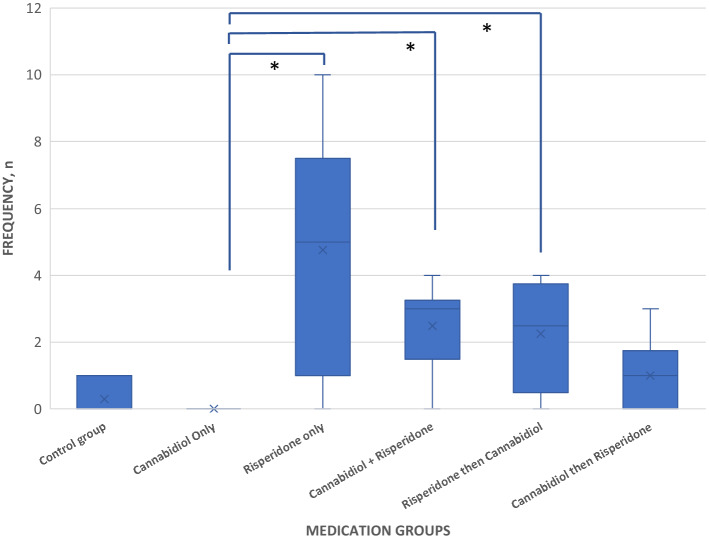
Vacuous chewing movements (VCM) across groups after the last dose of medications. CBD had effects on risperidone induced VCM. *Statistically significant difference.

After the last dose of pharmacological intervention, there was a statistically significant difference in VCM between the groups (F = 9.131, p < 0.001, df = 5). On post-hoc analysis, there were a significant difference between Groups D and B (p = 0.043), C and F (p = 0.003), C and A (0.000), C and B (p = 0.047), and E and B (p = 0.038) after the last dose of the medication. See Fig. 2.

#### Calculated open arm

There was no significant difference in the mean difference of the duration spent in the open arm of the elevated plus maze across the groups (F = 0.334, p = 0.890, df = 5).

#### Discrimination index

There was no significant difference in the mean difference of the discrimination index across the groups (F = 1.611, p = 0.178, df = 5).

#### Line crossing

There was no significant difference in the mean difference of locomotor activity across the groups (F = 2.262, p = 0.092, df = 5).

#### Rota rod

**Figure 3:**
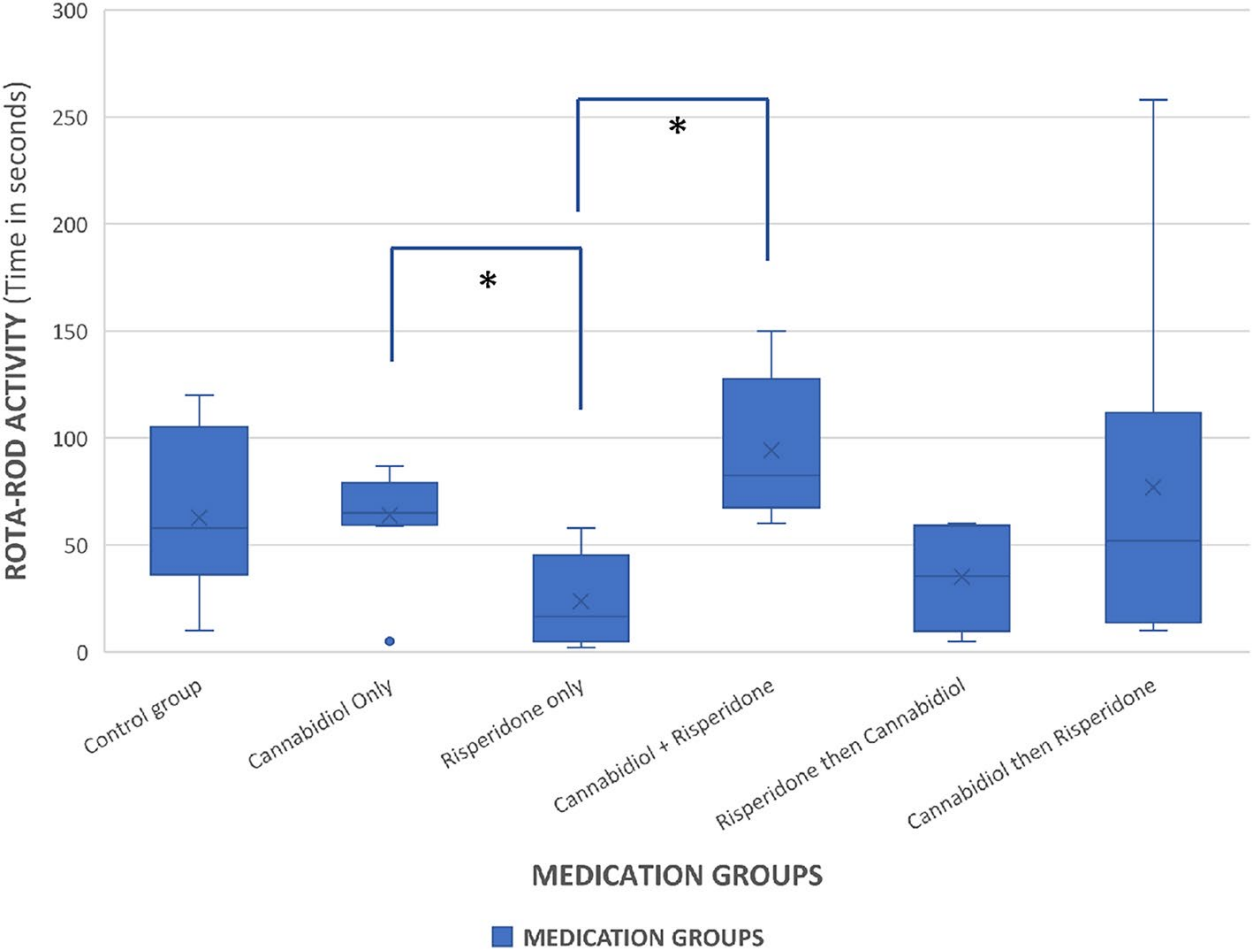
Inter-group comparison of the observed time spent on the treadmill in each group during the Rota-rod experiment. There was increase in time spent on the treadmill in Group B compared to the rest of the groups. *Statistically significant difference.

There was a statistically significant difference between the Rota Rod values between groups (F = 2.738, p = 0.031, df = 5). This difference was noted between Groups A and B (p = 0.019) and groups B and F (p = 0.027) (Fig. 3).

### Brain oxidative stress parameters

#### Brain GSH

There was a statistically significant difference in brain GSH between groups (F = 6.156, p < 0.001, df = 5). On post-hoc analysis, the differences were significant between Groups C and A (p = 0.001), E and A (p = 0.020), F and A (p = 0.000), and A and B (p = 0.003).

#### Brain SOD

There was a statistically significant difference in brain SOD between groups (F = 11.534, p < 0.001, df = 5). On post-hoc analysis, there were significant differences between Groups D and F (p = 0.006), D and A (p = 0.016), D and B (p = 0.012), C and F (p = 0.005), C and A (p = 0.012), C and B (p = 0.011), and F and A (p = 0.010).

#### Brain CAT

Significant differences were observed in the brain CAT values between groups (F = 13.870, p < 0.001, df = 5). On post-hoc analysis, these differences were observed between D and F (p = 0.002), D and A (p = 0.004), D and B (p = 0.002), C and F (p = 0.005), C and A (p = 0.008), C and B (p = 0.004), E and F (p = 0.047), E and B (p = 0.041), and A and B (p = 0.001).

#### Brain DPPH

Similarly, a significant difference was observed in the brain DPPH values between groups (F = 4.421, p = 0.004, df = 5). On post-hoc analysis, these differences were observed between Groups D and E (p = 0.033), D and A (p = 0.013), D and B (p = 0.000).

#### Brain NO

Values significantly differed between groups (F = 4.620, p = 0.006, df = 5). On post-hoc analysis, the differences were observed between Groups D and F (p = 0.041), D and B (p = 0.006), C and B (p = 0.002), A and B (p = 0.037).

#### Brain MDA

**Figure 4:**
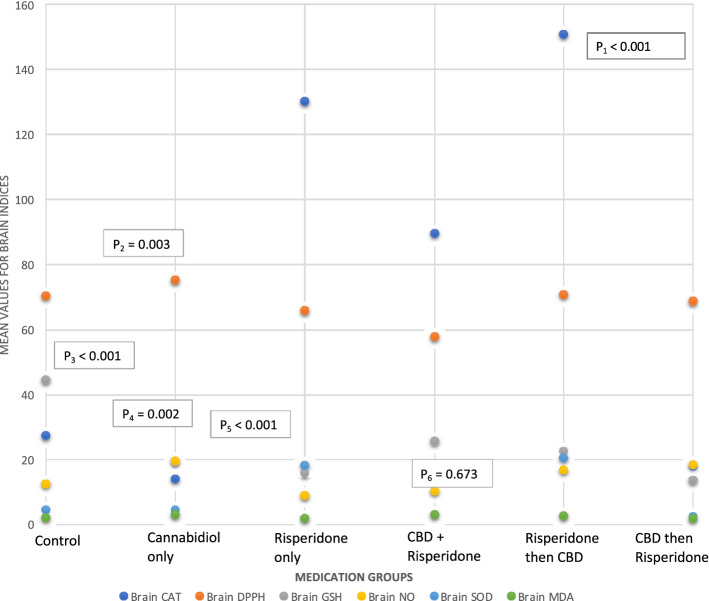
Inter-group comparison of assay of antioxidant indices assay after the last dose of medications. There were intergroup differences in the Brain’s GSH, SOD, CAT, SOD and DPPH results, but there were no intergroup differences in the brain’s MDA. Group 1: Control Group; Group 2: Cannabidiol only; Group 3: Risperidone only; Group 4: Cannabidiol + Risperidone; Group 5: Risperidone then Cannabidiol; Group 6: Cannabidiol then Risperidone. **P**_**1**_** = **P-value for brain CAT differences between groups; P_2_ = P-value for brain DPPH differences between groups; P_3_ = P-value for brain GSH differences between groups; P_4_ = P-value for brain NO differences between groups; P_5_ = P-value for brain SOD differences between groups; P_6_ = P-value for brain MDA differences between groups.

Brain MDA values did not significantly differ between groups (F = 0.617, p = 0.688, df = 5) (Fig. 4).

## Discussion

The present study is a continuation of our studies on the effectiveness of CBD in the treatment of symptoms of TD in an animal model. In this set of analyses, we focused on the synergistic effects of oral CBD and oral risperidone on weight, FBS, behavioural and oxidative stress parameters in the brain. We also assessed the effect of oral CBD on oral risperidone-induced VCM. We did not observe a significant increase in weight after the last dose of medication when risperidone alone and risperidone co-administered with CBD were compared. Concomitant administration of risperidone and CBD did not ameliorate VCM, unlike what we observed with haloperidol-induced VCM in our previous study^48^; however, there were distinct changes in antioxidant and pro-oxidant compounds, thus suggesting that both risperidone and CBD reduced oxidative stress in our experiments.

Moreover, our investigation did not reveal a significant change in weight when pre- and post-medication weights of all groups were compared, though association of risperidone with weight increase has been documented in animal and human studies^69–72^. Risperidone-induced weight gain may be associated with histamine H_1_ receptor antagonism, leptin resistance, and consequent disinhibition of food intake^73^. CBD has also been shown to decrease weight in animal studies^74^, but other studies contradict these observations^75,76^.

Comparison of FBS values across groups strongly suggests that oral CBD on its own can stabilize blood glucose. Most studies on the hypoglycaemic effects of CBD have reported similar findings, though experimental designs and doses varied^77–79^. Mechanisms of action may involve stimulation of insulin production by the beta-pancreatic cells and protective and regenerative effects on the beta cells^80,81^. When given concurrently with risperidone, CBD may not be effective as a hypoglycaemic agent, but CBD given after risperidone as in Group E (Risperidone before CBD) obviously reduced FBS and appeared to have similar effects as CBD alone, indicating that CBD may be effective in lowering blood sugar if given after the administration of risperidone. Results of CBD given before risperidone (Group F) were similar to those of risperidone alone, suggesting that CBD may not be effective in lowering blood sugar if given before the administration of risperidone. These findings require confirmation with a pharmacokinetic/pharmacodynamic (PK/PD) study. Meanwhile, risperidone has been shown to increase total and active ghrelin levels as well as leptin, and prolactin levels in some studies; and these are associated with increased appetite and metabolic dysfunction^72^, while CBD was shown to reduce appetite and insulin resistance by lowering resistin levels in some studies^81^.

In this study, risperidone alone (Group C) induced more VCM than all the groups, but this was only significant with CBD before risperidone, CBD alone, and the control group. We also observed that CBD administered concomitantly and after risperidone resulted in less VCM count than risperidone alone, though this was not significant. Our observations suggested that though CBD ameliorated the VCM induced by risperidone, and the effect was not quite pronounced, it may have a protective effect if administered before risperidone, as in Group F. This is contrary to what was observed with haloperidol-induced VCM in an earlier study, where CBD (5 mg/kg) administered concomitantly with haloperidol significantly reduced VCM^48^. We therefore propose that the aetiology and pathophysiology of risperidone-induced VCM may be different from that of haloperidol. Further investigations are needed on the aetiology and pathophysiology of risperidone-induced VCM.

In our model of motor coordination, balance, and grip strength, we observed that combinations of risperidone with CBD prevented the motor incoordination and increase in muscle tone associated with risperidone alone. The rats in the risperidone-alone group spent the least time on the treadmill, though this was only significant when compared with the group that received risperidone and CBD concomitantly (Group D), and the control (Group A). Risperidone is known to have a high affinity for D_2_ receptors and M_4_ receptors. D_2_ receptors are involved in EPS, while M_4_ receptors play a significant role in catalepsy, a condition where rodents have difficulty in changing externally-imposed posture^82,83^.

CBD is known to interact with 5HT_1A_ and 5HT_2A_ receptors subtypes in the basal ganglia to ameliorate the EPS induced by D_2_ receptor occupation^84,85^. CBD also influences phosphoinositide metabolism stimulated by activation of muscarinic receptors^86^. These factors may explain the effects of CBD in our experiment. To our knowledge, there are no published data on the effects of CBD on risperidone-induced movement disorders.

We could not confirm an anxiolytic effect for risperidone, either alone or in combination with CBD in our study using the elevated plus maze. This contrasts with a study which showed that risperidone at low dose induced an anxiolytic effect^87^. CBD at a higher dose than what we used in this study has reported anxiolytic effects^47,86^. We may not have observed an anxiolytic effect of different combinations of risperidone and CBD in our work because of the relatively low dose of CBD and high dose of risperidone. We expected the high dose of risperidone to affect the explorative activity and locomotion of the animals^87^ in the open field test because the receptor occupancy of risperidone at a high dose is above 60% and at this level, 5-HT_2_ blockade may not be protective against EPS with the development of parkinsonism and motor retardation^88^. At this dose however, we did not observe any differences when risperidone alone was compared with CBD only and the control groups, thus indicating that risperidone alone may not produce motor retardation or decrease locomotion at a high dose in rats. This suggests that 5-HT_2_ blockade may offer some protection.

In this study, there was no evidence of deterioration of cognitive function despite the high dose of risperidone. There was also no evidence for enhancement of cognitive function when risperidone was combined with CBD. Some clinical studies have observed an improvement in cognitive function in patients with schizophrenia on risperidone^89^. Results from animal studies indicate improved cognition with CBD alone^47^, but these results are contrasted by clinical studies that have found no clear clinical evidence of the positive benefits of CBD on cognition^75^. There are no published studies on the cognitive effects of combining risperidone and CBD.

### Effects of interventions on oxidative stress indices in the brain

Glutathione peroxidase (GPx) is the enzyme that catalyses the reaction between reduced GSH and hydrogen peroxide (H_2_O_2_) to form GSSG (glutathione disulfide), its oxidized form and water^90^, and elevated GSH therefore indicated less activity of GPx. The control group (Group A) had a significantly higher GSH level in the brain compared to other groups, with either CBD or risperidone alone, or in combination at different points, thus indicating that GPx is not active in the control. Lower GSH levels were observed in CBD and risperidone groups compared with the control, suggesting that these groups stimulated glutathione peroxidase production, leading to increased use of reduced GSH, which is converted to oxidised GSH (GSSG), and therefore lower GSH levels, compared to the control group.

SOD activities increased in the risperidone alone group (Group C), risperidone plus CBD (Group D), and risperidone before CBD (Group E) when compared with CBD before risperidone (Group F), CBD alone, and the control. These observations suggest increased antioxidant activities in the risperidone alone group (Group C), risperidone plus CBD (Group D), and risperidone before CBD (Group E) compared to others. CAT activities in the brain reflected a similar pattern except that risperidone administered before CBD produced higher activity than CBD before risperidone and CBD alone.

Though the effect of atypical antipsychotics on oxidative stress enzymes is still being explored, one study suggests atypical antipsychotics increase the expression of SOD1 gene leading to an increase in SOD^91^. We know that SOD acts as a first line of defence against oxidative stress by converting superoxide radicals to hydrogen peroxide, which is in turn converted to water and oxygen by catalase and glutathione peroxidase. An increase in SOD activity may indicate antioxidant potential or a compensatory mechanism^92,93^, depending on the level of pro-oxidants in the organ.

The lower level of GSH, coupled with the observed increased activity of SOD and CAT enzymes in risperidone alone, risperidone concomitantly administered with CBD, and risperidone before CBD groups suggests that both risperidone and CBD may have antioxidant effects. Meanwhile, CBD before risperidone antioxidant status (reduced SOD, CAT, and high GSH concentrations) compared to risperidone concomitantly administered with CBD and risperidone before CBD, suggests that CBD did not have a positive effect on oxidative stress when given before risperidone. We are not able to clearly explain our observation, which may be related to the ability of CBD to inhibit cytochrome P450 group of enzymes^49,94^ involved in the metabolism of antipsychotics and an increased concentration of risperidone to a level where it behaves like a typical antipsychotic^95^.

There were no significant group differences in MDA, a known marker of lipid peroxidation concentration in the brain, when all six groups were compared in this study. Another study also did not observe a change in lipid peroxidation in the brains of rats treated with risperidone after 45 and 90 days^25^. A group of investigators, however, observed that risperidone decreased MDA levels in rat brains suggesting that it could have antioxidant effects^28^. Relatedly, a clinical study claimed an increase in MDA levels in patients with schizophrenia, which was modified by atypical antipsychotics^92^. Some researchers have also observed a decrease in glutathione peroxidase (GPX) activity with an increase in MDA levels, suggesting an increase oxidative stress in schizophrenia patients on risperidone^96^. Our study concurs with other studies showing that risperidone has antioxidant properties^92^. This may explain our observation that MDA activity in risperidone-alone animals was not increased when compared with CBD alone, or any of the CBD combinations with risperidone.

This study also observed reduced NO production in the risperidone-alone (Group C), and risperidone given concomitantly with CBD (Group D) groups compared with the control and CBD-alone groups. Both risperidone and CBD have been shown to reduce inducible NO synthase, and NO production by some researchers^97–102^. Other studies that observed increased NO production in risperidone, however, contradict these reports^103–106^. In this study, risperidone appears to be an effective antioxidant, but risperidone and CBD given concomitantly is as effective as risperidone alone. CBD alone (Group B) appeared to be less effective in reducing NO level, compared to risperidone alone (Group C), and risperidone given concomitantly with CBD (Group D). We are not aware of any studies that have examined the antioxidant status of CBD combined with risperidone.

Our observations of the antioxidant effects of risperidone support our proposal that the aetiology and pathophysiology of risperidone-induced VCM may be different from that of haloperidol because risperidone had antioxidant effects in our study and did not induce oxidative stress, in contrast to haloperidol^55^. Earlier researchers also observed that sub-chronic treatment with risperidone did not increase the population of D_2_ receptors in the rat striatum, in contrast to the actions of haloperidol^107,108^. These observations suggest that oxidative stress has a role to play in the manifestation of movement disorders induced by haloperidol but may not be important in movement disorders produced by risperidone. The DPPH radical scavenging activities in the brain of all the groups were within the same range, but the risperidone plus CBD group (Group D) had fewer scavenging activities than the other groups. There is no known published study on DPPH radical scavenging activities in animals administered risperidone and CBD, but an in-vitro study used a DPPH assay to confirm the antioxidant status of risperidone^109^.

In summary, we found that CBD ameliorated the increase in FBS induced by risperidone if given before or after risperidone, and improved muscle incoordination and tone induced by risperidone, but it did not significantly reduce risperidone-induced VCM. CBD may, however, have a protective effect against VCM if given before risperidone. Concomitant or sequential administration of CBD and risperidone did not have any adverse effects on cognition or locomotion. Both CBD and risperidone therefore exhibited antioxidant properties.

## Limitations

First, 21 days of administration of CBD, and distilled water in the control group instead of 28 days is an important limitation of this study. All treatments should have the same duration; however, these data were drawn from a larger study that included haloperidol that was administered for 21 days. The IRB at the institute at which the experiments were conducted considered the same control to be adequate for the study involving haloperidol and risperidone.

Second, the animal model of TD used in this study may not be specific enough for TD, and may closely resemble EPS (e.g. Parkinsonian tremor, dystonia), even though only VCM that persisted after the 8th day was used in this study to reduce the probability that what was observed is EPS. Furthermore, the experimental paradigm used in this study closely resembles that of Bishnoi and Boparai^110^, and Marchese^55^.

Another limitation observed in this study is that automated behavioural apparatus would have produced more precise and less biased measurements, but financial constraints prevented us from procuring this apparatus. It was not possible to measure oxidative stress indices in specific regions of the brain due to technical difficulties.

## Conclusion

Our results suggest that a combination drug formulation of risperidone and CBD in clinical practice may have less metabolic dysregulation and extrapyramidal side effects than with risperidone alone, without producing cognitive impairments as seen with the olanzapine and samidorphan^111^ combination. Such a combination may also improve the overall safety and tolerability of risperidone. Clinical studies will be required to prove the efficacy of risperidone and CBD combination. Pharmacokinetic and pharmacodynamic studies in both humans and animals are also necessary to further study risperidone and CBD drug-drug interactions.
